# What happens at two? Immunisation stakeholders’ perspectives on factors influencing sub-optimal childhood vaccine uptake for toddlers in regional and remote Western Australia

**DOI:** 10.1186/s12913-024-11371-8

**Published:** 2024-08-22

**Authors:** Samantha J. Carlson, Sian Tomkinson, Adam Hannah, Katie Attwell

**Affiliations:** 1 Wesfarmers Centre of Vaccines and Infectious Diseases, Telethon Kids Institute, 15 Hospital Avenue, Nedlands, Perth, 6009 WA Australia; 2 School of Social Sciences, The University of Western Australia, Perth, WA Australia; 3 School of Political Science and International Studies, The University of Queensland, Queensland, Australia

**Keywords:** Vaccination, Children, Rural health services, Health behavior, Focus groups

## Abstract

**Background:**

In Western Australia (WA), children aged 24 months living regionally or remotely (non-urban) have suboptimal vaccine uptake. As there has not yet been a systematic approach to understanding the facilitators and barriers to childhood vaccination in regional and remote WA, this study aimed to understand the views of key immunisation stakeholders regarding barriers and solutions.

**Methods:**

Drawing on the World Health Organization’s “Tailoring Immunization Programmes” approach, we undertook a qualitative study with three forms of data collection: (1) semi-structured interviews with immunisation experts within Australia’s immunisation system, (2) a semi-structured focus group with immunisation coordinators and health workers working in regional or remote WA, and (3) member checking with senior staff from WA Health. Data from the interviews and focus group was deductively analysed using the Capability-Opportunity-Motivation-Behaviour (COM-B) model on NVivo 20.

**Results:**

There was no clear consensus on the typical under-vaccinated child in country WA. A range of barriers were identified: lack of awareness of the vaccine schedule, difficult access to vaccination services, a shortage in a workforce able to have meaningful conversations with vaccine hesitant parents, ineffective reminder systems, and the rapid spread of misinformation. Participants described previous efforts used to improve vaccine uptake, and felt the following would improve uptake: better access to vaccine clinics, building capacity of Aboriginal Health Workers, and vaccine reminders.

**Conclusion:**

This is the first time the facilitators and barriers to routine childhood vaccine uptake in country WA has been explored. Addressing some of these barriers may see an increase in uptake.

## Background

Sub-optimal childhood vaccination coverage can cause illness and death. Falling uptake and corresponding outbreaks of vaccine preventable disease are recently evident in Europe [1] and have occurred in Australia, including Western Australia (WA) [2]. Of particular concern was a 2019 measles outbreak in WA, which was the worst seen in two decades [3].

Australia’s aspirational childhood vaccine coverage target is 95% [4]. These targets are assessed in the following ways [5, 6]:

1. One-year-old cohort (assessed among children aged 12 to < 15 months): the child must have received all the vaccines due at six months of age (3x combination vaccines covering diphtheria, tetanus, pertussis, hepatitis B, polio, and *Haemophilus influenzae* type B, 2x pneumococcal), as per Australia’s National Immunisation Program (NIP) [7].
2. Two-year old-cohort (assessed among children aged 24 to < 27 months): the child must have received the vaccines due at six months (described above), 12 months (1x Meningococcal ACWY, 1x combination vaccine covering measles, mumps, rubella, and 1x pneumococcal), and 18 months (1x *Haemophilus influenzae* type B, 1x combination vaccine covering measles, mumps, rubella, and varicella, and 1x combination vaccine covering diphtheria, tetanus and pertussis), as per Australia’s NIP.
3. Five-year-old cohort (assessed among children aged 60 to < 63 months): the child must have received the vaccines due at 48 months (1x combination vaccine covering diphtheria, tetanus, pertussis, and polio), as per Australia’s NIP.

Australia-wide, the most recent annual data (calculated in December 2023) illustrates that 93.16% of children in the one-year-old cohort were fully vaccinated, 91.24% of children in the two-year-old cohort were fully vaccinated, and 93.93% of children in the five-year-old cohort were fully vaccinated [8]. The coverage in all cohorts across Australia has declined since 2020 [9].

There are some success stories of vaccine uptake in WA, including overall uptake in all children aged < 60 months increasing from 91.7% in 2017 to 93.1% in 2021 [10] and vaccine coverage in Aboriginal children aged 60 months being above the 95% target (Table 1) [10]. Improvements occurred after changes to the federal policy linking vaccination requirements to government-funded financial assistance for families (removing personal belief exemptions to vaccination via a policy termed ‘No Jab, No Pay’) [11].

However, there is still room for improving vaccine coverage in WA. This is particularly evident in children aged 24 months, for whom uptake declines from 12 months in both Aboriginal and non-Aboriginal children, and in both regional/remote (non-urban) WA and Perth (Table 1– reporting on coverage assessed at time of study). Though this decline for 24-month-olds is also seen nationally [8], it is important to identify local drivers and solutions.

**Table 1:** 2021 childhood vaccine coverage in various age groups in Western Australia, by aboriginality

	Aboriginal			Non-Aboriginal		
	1-year-old cohort (%)	2-year-old cohort (%)	5-year-old cohort (%)	1-year-old cohort (%)	2-year-old cohort (%)	5-year-old cohort (%)
Regional/remote	87.3	**86.8**	95.1	92.9	**90.9**	93.1
Perth	86.8	**84.7**	95.1	94.8	**92.5**	93.7
All Western Australia	87.0	**85.7**	95.1	94.5	**92.3**	93.6

Data source: [10]

Although remoteness was found not to affect vaccine uptake for Western Australian children in a recent study [12], regional health services would nevertheless benefit from understanding reasons for sub-optimal vaccine uptake in their regions, particularly in children aged 24-months, and any local solutions attempted or attained by staff working on the ground. The WA Country Health Service (WACHS) is the public health service provider/public health unit for people who live in regional and remote WA, and is one of several public and private entities that administer National Immunisation Program (NIP) vaccinations in WA’s seven non-urban regions: the Kimberley, Pilbara, Midwest, Wheatbelt, Goldfields, Southwest and Great Southern. WACHS takes a lead role in seeking to increase the vaccine coverage rate to 95% in these regions, which has not yet been reached in any region among the 2 year old cohort. Since 2019, the Pilbara region has had the lowest vaccine uptake at 24 months, with 82.15% of children in the region fully vaccinated as at September 2023 [13].

Experts often classify the overarching barriers to vaccination as either “access” or “hesitancy” [14]. Both are evident in WA’s regions. Small pockets in the Southwest and Great Southern regions of WA contain vaccine hesitant and refusing communities [15]. On the access front, parents from the Southwest have previously reported a desire for after-hours and weekend appointments, as well as reminders to help them keep up to date [15]. The complexity of vaccine governance in Australia – which includes local, state and national stakeholders – poses further challenges. There may have also been historic issues regarding wastage and cold chain processes [16]. Further, the vaccine schedule and policies often change: doctors in the Southwest have indicated a desire for face-to-face updates as well as print resources on immunisation [15].

Another possible explanation for the low coverage rate measured for 24-month-olds is that the data may be inaccurate. A 2014–2015 survey found that data on the Australian Childhood Immunisation Register (ACIR) was often incorrect, with vaccinations not being recorded [16]. The Australian Immunisation Register (AIR), which expanded the ACIR into a whole-of-life immunisation register, has also been found to contain inaccurate data, both due to users entering information incorrectly and compatibility issues with software used in medical practices [17]. A further issue is that the algorithm used to measure “fully vaccinated” status changed in 2016 and 2018 [8], possibly affecting coverage rates independent of changes in the number of children actually getting vaccinated [18, 19].

Hence there are numerous factors that could explain the sub-optimal immunisation coverage rates of 24-27-month-olds in regional WA. This situation is not unique, and reflects increasing concern internationally regarding routine vaccination, which has also been impacted by the COVID-19 pandemic [20]. As there has not yet been a systematic approach to understanding the facilitators and barriers to childhood vaccination in regional and remote WA, this study aimed to understand what key immunisation stakeholders believe the barriers and solutions are. This project has not only local but national and international benefits, as some barriers to vaccination in regional WA are likely shared by other jurisdictions.

## Methods

### Data collection and participant recruitment

Drawing on the WHO’s “Tailoring Immunization Programmes” approach [21], we undertook a qualitative study in 2021 as part of a broader project seeking to understand and address sub-optimal childhood vaccination coverage in regional and remote WA. We used three forms of data collection:

#### Semi—structured interviews with experts in Australia’s immunisation system

The second author undertook semi-structured interviews with key informants, who provided insight into potential reasons for why coverage rates are low for 24-month-olds in regional areas. Informants were from the National Centre for Immunisation Research and Surveillance (NCIRS), who are experts in the recording and processing of vaccination data in Australia, and the WA Primary Health Alliance (WAPHA), who support, plan, fund, monitor, and evaluate primary health care services in WA. Based on our expert knowledge of the WA and Australian vaccination ecosystem, we approached these informants in writing and asked them to participate.

Informants were asked about their organisation’s role in immunisation, where they access coverage data and their understanding of the ‘full vaccinated’ definition, their understanding of reasons for suboptimal vaccine coverage in regional/remote WA, and any challenges they are aware of in delivering immunisation programs in regional/remote WA. Interviews were conducted via videoconferencing software, Zoom, and were audio-recorded with participant’s consent.

#### Focus groups with WA-based immunisation coordinators and health workers

We undertook semi-structured focus groups with WA Health participants through an online workshop format. The workshop was planned to include immunisation coordinators and health workers from each region, WACHS immunisation program leads, and a representative from the state’s Communicable Diseases Control Directorate (CDCD). Senior WACHS staff provided the research team with the names and contact details of the Regional Immunisation Coordinator (RIC) from each of the 7 regions. The research team invited each RIC via email to participate. We also utilised the snowballing recruitment method by asking the RIC to nominate an additional key health worker/immunisation provider from within their region.

The three-hour recorded workshop was run via Zoom, in response to the workloads of healthcare workers located in geographically dispersed areas. The research team drew on their expertise in facilitating face-to-face focus groups and online pedagogy to mitigate dominant or quiet participants. To avoid participants becoming uncomfortable speaking in front of each other, we separated those from the same region.

**Fig. 1:**
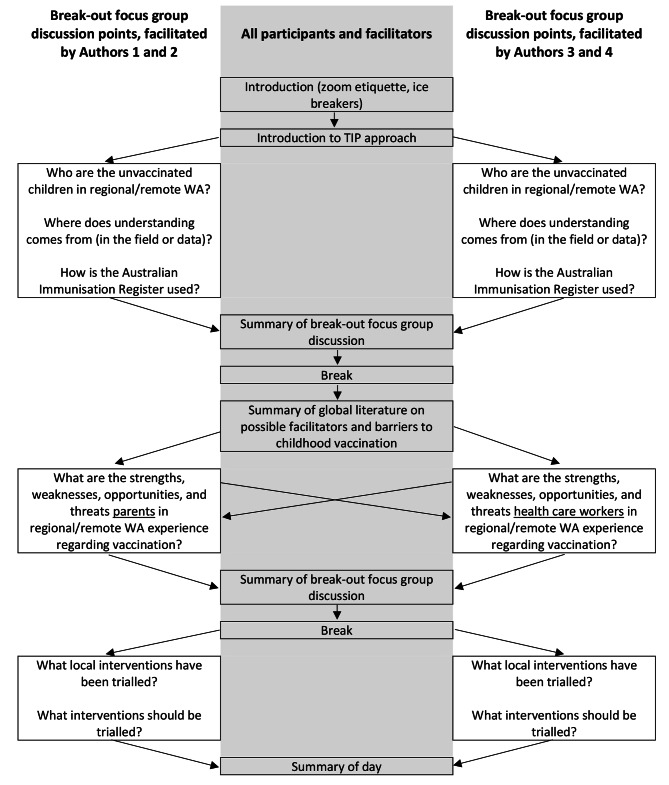
Structure of workshop with WA Health staff Note: direction of arrows indicates the way in which participants experienced the focus group, where they joined the main focus group, and then broke out into smaller focus groups, then re-joined main focus group

The workshop broadly followed the guidance given in the Tailoring Immunisation Programs (TIP) manual [21]; we also consulted with TIP expert, Dr Cath Jackson to tailor it to our stakeholders. TIP was developed by the World Health Orgnization Regional Office For Europe which the intention of providing those working in immunisation with the tools to (1) identify suboptimally vaccinated cohorts, (2) understand the facilitators and barriers to vaccination, and (3) design interventions that target the identified barriers [21];. The structure of the workshop is presented in Fig. 1, in which participants were split into 2 focus groups for the semi-structured discussions, and brought back together for a summary discussion. Within the focus group breakout sessions, participants were asked about:

1. Their knowledge of vaccine coverage in WA, and the characteristics of under-vaccinated children
2. Their vaccine coverage data sources
3. The faciliators and barriers for healthcare workers to administer vaccines to children in regional/remote WA
4. The facilitators and barriers that parents/carers in regional/remote WA experience regarding childhood vaccination
5. Their knowledge of, and potential involvement in, the implementation of local interventions to improve childhood vaccination coverage rates

#### Validation of data interpretation with WA Health staff

We discussed findings from the formal focus groups and semi-structured interviews in meetings with senior staff from WA Health, who were able to provide context and explanations for comments made during the focus group. Meetings were not audio-recorded, and were not included in the formal data analysis. Rather, the research team were able to adapt the way in which they interpreted the data based on the in-depth insights provided by those involved in the system.

### Data analysis

All data from the interviews (summaries and noteworthy quotes written by interviewer, ST) and workshop (verbatim transcripts) were thematically analysed using NVivo 20. Bringing this data together allowed us to triangulate our analysis and identify and resolve any discrepancies for follow up in meetings with WA Health, as described above. The first and second authors undertook mostly deductive coding following the TIP adaption of Capability-Opportunity-Motivation-Behaviour (COM-B) model [21, 22]. **Capability** refers to the knowledge and skillset an individual possesses to undertake the behaviour, **opportunity** refers to external physical and social factors that influence behaviour, and **motivation** refers to the cognitive processes that subsequently influence behaviour. All these factors interact for the behaviour to occur (or not). The authors used the subthemes outlined in the TIP manual under each COM-B category as their coding framework [21]. Additionally, some inductive coding of the data that did not fit within COM-B categories was undertaken. Differences between participant types (i.e. a key informant from an interview versus an immunisation coordinator/provider from the workshop) were not explored in the analysis – however, if there were noteworthy findings from particular regions, these are highlighted in a way that also respect’s the participant’s anonymity.

## Results

We interviewed four key informants: two from NCIRS and two from WAPHA. The workshop included 16 participants from WA Health; combined, we were able to hear from participants in all but one of WA’s 7 regions. Due to the high workload of regional health staff during COVID-19, in some regions we could not speak to workers in all roles.

Here we present findings on participants’ perceptions of the attributes of under-vaccinated children in WA country regions, their views on the systems in place to monitor vaccine coverage, and the facilitators and barriers to childhood vaccination in WA country regions. We include illustrative quotes with pseudonyms attached.

### Identifying the under-vaccinated children in WA country regions

There was no clear consensus on the typical under-vaccinated child in country regions, but Dianna’s statement reflected the factors at play:

*I would say that the group is so wide and diverse you can’t characterise them. You’ve got the educated*,* you’ve got the non-educated*,* you’ve got the vaccine refusers*,* you’ve got the internet explorers*,* you’ve got the busy mums*,* you’ve got the underprivileged people who are just doing it really tough*,* you’ve got the overseas immigrants who believe that [the] Immigration [Department] have sorted out all the vaccination problems when they come to Australia. Just so many diverse reasons for people becoming overdue; there’s no particular characteristic at all –* Dianna, workshop participant.

Some participants considered that Aboriginal and Torres Strait Islander children were an under-vaccinated cohort in regions such as the Kimberley and the Wheatbelt. Jeff, a key informant, however, said:

*In general*,* there’s not much vaccine hesitancy in Aboriginal communities*,* there’s actually a high level of support. The problem is access and timeliness*.

Some participants were quick to point out that given the small population in remote towns, the few unvaccinated children heavily skew the overall vaccine uptake percentage for that cohort.

### Systems in place to monitor vaccine coverage

AIR data provides WA Health staff with the ability to generate reports on children who are due or overdue for vaccination. Some participants were aware of and used one of two types of these reports. However, neither appear to sufficiently cater to the needs of health services: many participants described the poor data transfer success rates between immunisation providers’ clinical software and the AIR. Taylor (key informant) said that whenever there is a change to the vaccine schedule, the software that immunisation providers use needs to update, which can often take up to 6 months. This results in data errors.

Frustration emerged among many workshop participants at the insufficiently clear lines of responsibility for data management and uncertainty regarding which government agencies possess authority to provide solutions. The present malaise, according to workshop participants, sees some children being incorrectly recorded as unvaccinated and some even being revaccinated when their original doses are not registered on the AIR.

Many participants indicated that many General Practice clinics put incorrect information into AIR, which then must be manually changed. They reported spending considerable time “cleaning” the data to make sure it accurately reflects what they understand to be the reality of a child’s immunisation status. This involves phoning parents and General Practice clinics.

Most participants said they also used high-level reports to understand which immunisation providers are accessing the reports that display children who are due or overdue for a vaccination. If providers are not using the reports, participants will often reach out to the provider to investigate and explain how to download and interpret the report. Roselyn (key informant) said this process often identifies children who have relocated overseas or interstate.

Another reason, according to participants, that the overdue reports are less effective in identifying parents who may respond to outreach is that the ability to register a parent on the AIR as a ‘conscientious objector’ (those with a personal belief exemption) was removed in 2016, when vaccine refusers were denied access to federal financial entitlements. Approaches to encourage an objector family to vaccinate can cause conflict and friction between families and immunisation providers. One region created their own database to keep abreast of such families:

*We…keep a little running spreadsheet of people that had nominated not to vaccinate so we don’t accidentally recall them again…it has been very nasty… If we could be advised prior to*,* that a family has elected not to vaccinate their children*,* that would help*,* because it always is a delicate conversation talking to a parent…You can almost feel the aggression within their voices*,* “How dare you be confronting me about this*,* I’ve made my personal decision about it.”-* Gina, workshop participant.

One workshop participant reflected that the rigid structure of AIR was not compatible with the often-fluid lives of migrants and refugees. The Register’s helpdesk could be unhelpful, including with regard to removing non-resident children who were distorting the numbers of unvaccinated in a region.

WA Country Health Service holds quarterly business performance meetings with each region and these meetings include discussion on immunisation rates, where co-ordinators “*have to explain themselves as to why their rates are below the required rate*” (Sue). Yet even these discussions are mired by discrepancies between AIR data and the internal WACHS dashboard:

*When I have to respond to the dashboard data*,* sometimes it seems to be a little out of date… If we’ve just received some new data*,* sometimes we’ve improved …*,* and I think*,* “We’re not that bad*,* we did quite well this quarter!” I think sometimes there’s a bit of a delay in the way it filters through. I mean*,* I don’t know where that dashboard data comes from*,* or who pulls it*,* or who writes up the report*,* but sometimes there seems to be a mismatch*,* from where I sit* – Dianna, workshop participant.

WA Health staff later explained in a validation meeting that this is a result of different reporting timeframes, in which AIR data labels the current data as the previous quarter (of a year).

Finally, there was some discussion about the impact of ‘the algorithm’ changing. Workshop participants and key informants were of the view that changes to the definition of ‘fully vaccinated’ is not the sole reason for lower vaccine uptake in 2-year-old children:

*we’re talking about the calculation [changing]…five or six years ago. We can no longer use that as the [excuse]. I would say that it’s quite irrelevant now because so much time has elapsed… –* Dianna, workshop participant.

### What facilitates or prevents vaccine uptake in regional WA?

We explored participant’s views on what facilitates and prevents vaccination in remote and regional WA, in terms of parents’ capability, opportunity and motivation to vaccinate. We focused most on opportunity, since this is the modifiable “service delivery” aspect of vaccine uptake that is the core business of our participants as government immunisation providers. However, we reflect on all aspects of the model.

#### Capability

##### Knowledge of vaccines

Some participants said some parents, such as those born overseas, are unaware of the vaccine schedule in WA and the accessibility of different clinics:

*There are [general practitioners (GPs)] who are open up to 8 o’clock*,* so there is after hours [access]… It’s just making sure that they are aware of the service –* Paula, workshop participant.

*There’s many a time that I’ve had somebody that we’ve contacted in some way*,* and they just didn’t know that we were here to vax* – Camille, workshop participant.

Conversely, some participants said a minority of parents are extremely aware of the schedule and question the appropriateness of NIP vaccines in various ways. Participants discussed parents who talked (erroneously) about vaccines causing autism and containing mercury.

#### Opportunity

##### Access to vaccination services

Participants said that parents in regional and remote WA can access vaccines through public clinics run by WA Country Health Services on scheduled days and times, often at community centres, in which immunisation providers (usually nurses), may be required to travel from one small town in the morning to another in the afternoon. Workshop participants also noted that parents can also access vaccines more regularly through private providers, such as General Practice clinics and Aboriginal Medical Services, though this varies depending on the region. Some variability in providers seems to be due to parent preferences; for example, there is a “*group of women that have elected to take their immunisation and their child health services through GPs*,* not through [WA Health] staff*” (Gina, workshop participant).

However, workshop participants discussed how state government health providers (rather than private practice providers) are better able to navigate complex immunisation schedules, such as when a child needs a catch-up schedule. In these circumstances, WA Health staff may encounter other providers who are unsure how to proceed, or encourage those providers to refer the families to the Health Department:

*A lot of my GP practices will not do overdues. They actually flatly tell me they will not do overdues because they don’t know how to do them*,* even though I keep going*,* and saying*,* “ring me*,*” and give support*,* so it’s quite hard –* Beth, workshop participant.

Workshop participants described how numerous regions provide home visits to administer vaccines (a practice known as domiciliary vaccination). However, some participants were not comfortable with this practice. Some described risks to personal safety, such as verbal abuse and threats from parents, or encountering dogs with histories of biting people. Many also considered it resource-intensive:

*it always comes down to staff capacity at the time so it’s certainly not extensive*,* it is only ever tailored to people of particular need –* Dianna, workshop participant.

Some participants also regarded home visits as a “*retrograde step*” when families had the means to attend clinics, such as having “*four or five or six cars…but they can’t come in for their immunisations*.” They preferred to empower and activate parents by finding other solutions to get them to visit a clinic:

*We do a lot of home visiting*,* a lot of phone calling*,* a lot of SMSing*,* use of taxi vouchers for people that say they haven’t got money and they can’t get in. We’ve got a dedicated government vehicle that is set up with childcare seats… We do opportunistic immunisations outside scheduled clinic times*,* I do them myself*,* so we try and do that. We also connect with a local Aboriginal medical service and use their bus service to pick people up and bring people in. –* Gina, workshop participant.

Some participants noted that not all regions have a vehicle to bring families to a clinic, and in some regions walking is not an option for many given the extreme temperatures that WA experiences. Public transport also is not an option for many in regional/remote locations, leaving many families without a way to get to a clinic:

*Most of our people have to drive*,* walk*,* cycle. You know*,* most of the areas don’t have a public transport system. Some areas do have a public transport system*,* but it’s whether our clinics are…close by –* Sue, workshop participant.

##### Availability of vaccination services

Participants across multiple regions believed that parents of unvaccinated children are unable to access vaccines at a time that is most convenient for them. This problem was exacerbated by COVID-19. Social distancing requirements removed the ability to walk-in for an unscheduled vaccine, which, according to workshop participants, particularly affected Aboriginal families. Meanwhile, routine immunisation staff were being redeployed for COVID-19 vaccinations.

*We used to do more numbers ‘cause it was a walk-in clinic. Now with COVID*,* with the social distancing*,* it has to be by appointment only…Pre-COVID we were seeing larger numbers of people come in…[I see] more now our non-Aboriginal [clients]*,* because they have a phone to ring [us] to make appointments* – Melinda, workshop participant.

However, some workshop participants pointed out that barriers to accessing clinics were apparent before COVID-19, too. For example, standard clinic hours are difficult for those who do Fly In, Fly Out (FIFO) and shift work, or if both parents want or need to attend the appointment or have work commitments. The after-hours clinic in the one region is mostly attended by:

*…mums who don’t have driver’s licenses where they rely upon their partner*,* so they’ve got to wait for their partner to finish work…or the mother wants the father to be involved with all the kids and appointments*,* so we wait for dad to come home from work* – Nellie, workshop participant.

In that same region, some parents are also able to access vaccines at a time that suits them, rather than following a strict vaccine schedule that is not compatible with living on a large farm:

*We have clients who actually live on stations*,* hundred*,* two hundred*,* three hundred [kilometres] out of town*,* and we just coordinate*,* it’s a one stop shop. Come in: vaccination*,* shopping*,* groceries*,* child health checks*,* we coordinate it in to work around the family…So their vaccinations might be a little bit out or a couple of weeks out*,* but it’s about the whole family environment so they don’t have to do trip after trip.* – Nellie, workshop participant.

Many participants also strongly believed that the major barrier to vaccination for two-year-olds is the change in the timing of the routine child health/development checks. Until mid-2017, these checks coincided with the immunisation schedule and children had appointments at 18 months, providing health care workers with the opportunity to deliver vaccines concurrently. However, the 18-month check was replaced by a 24-month check.

*Now we only see the kids at 12 months and then again at 2 years and… the 2-year-old check can be done any time in a child that two-year period so*,* you know*,* 364 days. So consequently*,* by the time you see them they could’ve already [become] overdue* – Sue, workshop participant.

WA Health staff explained in a separate validation meeting we held with them that the Universal Child Health Schedule was revised in 2016 and implemented from 1 July 2017, following a review of evidence by Prof Karen Edmonds. Any subsequent changes to the schedule would be based on evidence and be resourced appropriately.

##### Affordability of vaccination services

A suggested barrier for many refugee and migrant families was that vaccines on the NIP are technically only free for those with a Medicare card and citizenship or permanent residency. General practice clinics charge families on a visa for vaccines, while WA Health clinics do not:

*If they’re on a visa*,* you know*,* they ring up [a General Practice clinic]*,* they will get told more than likely that … they’ll have to pay … But if they come into a Population Health Centre [run by WA Health]*,* we don’t ever ask if they’re on Medicare. I know we have to ask for their Medicare details and whatever*,* but if they don’t have them*,* we don’t turn anybody away* – Sue, workshop participant.

##### Regulation and legislation

Many participants regarded ‘No Jab, No Pay’ as prompting some disadvantaged families with competing priorities to vaccinate their overdue children. However, several caveats were also discussed. First, participants specified that the policy does not encourage timely vaccination. Rather, parents are prompted by a physical letter indicating their child is overdue and threatening the cessation of welfare payments. Second, high-income families not reliant on this particular benefit were generally not prompted nor “*bothered*” by the policy. Finally, one participant speculated that for some families receiving other welfare payments, such as unemployment or parenting payments (both of which are much larger than the payments targeted by ‘No Jab, No Pay’), there was not necessarily a strong additional incentive for parents to vaccinate.

Some participants also commented on the effectiveness of immunisation requirements for childcare enrolment in WA. Some participants pointed out that keeping up-to-date with vaccines is not a requirement for *staying* enrolled in childcare. Dianna, a workshop participant, described outreach strategies of poster and business card dissemination at childcare centres encouraging parents to speak to an immunisation expert within WA Health about their vaccine concerns if they were unable to enrol their child due to No Jab No Play:

*[So] the childcare manager will say “we’ve got some help for you*,* you’ll be able to talk to an expert in the field*,* here’s a business card*,* get in touch when you want to.”*

##### Skillset and confidence of vaccinators

While “*everybody who works within Population Health understands that [immunisation] is an important thing that we do*,” (Martha, workshop participant), there are areas in which participants felt the system inhibited immunisation providers providing top service to regional families. This was mostly related to the skillset and confidence of vaccinators, and staff shortages (described later), which impact on the timely vaccination of children.

Some participants believed vaccine uptake is best when the immunisation provider displays confidence, which then flows to parents:

*I think you need to be a confident vaccinator. You need to know your schedule well*,* you need to know why you’re giving what vaccine*,* and you have to be able to – by the way you engage – develop a rapport and a confidence with your client group. … And on top of that list is if you can get an excellent health worker or Indigenous health officer working with you*,* that’s 90% of your problems fixed –* Gina, workshop participant.

Other participants regarded immunisation providers in their region to be ill-equipped to converse with vaccine hesitant parents. Depending on the region, workshop participants said some providers offer hesitant parents the direct phone number to their regional immunisation coordinator to have this conversation, or the regional immunisation coordinator dials in to the consultation between the vaccine hesitant parent and the immunisation provider.

It was widely acknowledged by workshop participants that parents need to be able to trust in the service and their immunisation provider – this seemed to occur more when immunisation providers have been part of the community for many years, and show respect to different views and cultures. A major barrier identified by participants was the fact Aboriginal Health Practitioners (AHPs), at the time of data collection, could not administer vaccines. In later discussions with WA Health staff, it was explained that in some other Australian jurisdictions, AHPs appointed at a more senior level in government health departments, are licensed to administer vaccines.

*…we need more Aboriginal health … practitioners to be able to assist with the delivery of the vaccination program*,* especially for regional areas where there are large numbers of Aboriginal population… [WA Health] needs to endorse Aboriginal health practitioners to be able to vaccinate…Our Aboriginal Health Workers deliver the EACHS [Enhanced Aboriginal Child Health Schedule] program*,* they do a lot of input*,* they do a lot of education on parents*,* you know*,* they go to the homes of our population especially here…Where they go and talk to the parents and bring them in for their immunisations*,* they’re doing all the work*,* and then we come along and jab them*,* so why can’t we let them do the jabbing? – Melinda*, workshop participant.

The WA Health Department had partnered with the state’s peak body for Aboriginal controlled health services to develop a two-week course for Aboriginal Health Workers (AHWs) and AHPs to help them promote and administer vaccinations under the supervision of an immunisation provider [23]. Participants from some regions reflected on the fact that AHWs in their regions had completed this course but still lacked confidence to administer vaccines. They believed this was due to a local adverse event:

*At [our local Aboriginal health service]…their Aboriginal Health Workers have all got their vaccination certificates. But it was*,* like*,* a task they did but they never followed it through*,* ‘cause they never gave the confidence to vaccinate…They had a child that had a true anaphylaxis*,* an 8-year-old from the flu [vaccine]*,* it just gave them big fear…There’s probably about six of their health workers that went and actually did the course…and they never vaccinated –* Debra, workshop participant.

Participants also commented on an overall lack of confidence among some external providers:

*I think it’s also sometimes a confidence and a competency issue. I found that especially with GP practice nurses*,* that a lot of them won’t do overdues…because it’s a confidence*,* competence possibly*,* issue*,* and they don’t have people on the ground and training specifically for them –* Beth, workshop participant.

##### Staff shortages

According to workshop participants, a shortage in immunisation providers appears to be affecting vaccine access in regional and remote WA, which has resulted in parents walking into clinics for ad-hoc vaccination and being turned away, or being told not that the service is unavailable during a follow-up phone call.

*I phone them up and say “hey…Jimmy’s overdue for vaccination.” [The parent says] “oh can I come in now?” Unfortunately*,* I can’t offer them an appointment now and then –* Camille, workshop participant.

Staff shortages also seemed to be exacerbated by the demands of the COVID-19 vaccine rollout:

*If I’m having problems with my overdue reports*,* it’s indicative that there’s staffing problems in that district and it’s the flow on effect…Anecdotally we’re seeing so much burnout now with the COVID stuff impacting on normal service delivery*,* people are just not getting the services –* Beth, workshop participant.

##### Reminders

As one key informant noted, “*rock solid reminder systems*” are vital to encourage parents to keep their details and vaccinations up to date. Many workshop participants described how their region sends an SMS reminder about vaccination, and offer appointments either at a WA Health clinic or provide the details for a local General Practice clinic. However, this can result in requests to vaccinate immediately, which cannot be met by the WA Health clinic. SMS reminders seem to be the most utilised reminder form, but some clinics also make phone calls. One workshop participant, however, said they do not have the time to remind overdue parents as they are too busy vaccinating:

*I’m literally the little lamb on the ground running around nonstop*,* I don’t have time to even do recalls for immunisations*,* I’m concentrating on the babies*,* and then as I see them I will vaccinate when they come and see me without them having to line up or make appointments at vaccination clinics*,* so I’m actively trying to get them done*,* saves having double appointments –* Debra, workshop participant.

It was acknowledged by some participants that people who live in remote WA may not receive SMS reminders from WA Health if they don’t have reliable and working mobile phones. Parents also receive letters from the Federal Government, but Veronica, a workshop participant, added, “W*e don’t [all] have mailboxes…people just don’t get their mail out here…they’re moving constantly.”* One regional WA Health office also sends parents of children due or overdue for vaccines via registered mail; registering the mail helps the WA Health staff to know whether it arrived.

##### Availability and accuracy of information

The first formal pathway for discussing vaccination once families leave the maternity care system becomes the role of Child Health Nurses. These WA Health employees provide parents with information about the schedule, where vaccines can be received, and – in one region – book the child in for their 2-month vaccines during a conversation usually held in the child’s first two weeks of life. Audrey explained that at her site, Child Health Nurses themselves usually cannot administer vaccines at the Child Health appointments as their clinics usually are not equipped with immunisation fridges. A separate validation meeting with WA Health staff identified that these fridges are expensive and need to be monitored and stocked, which significantly increases workloads of already busy staff.

In the same region where Child Health Nurses assisted families with vaccine bookings, if families are “*showing a negative view on vaccination*,” the Child Health Nurse will refer them to websites with further information, or to an immunisation coordinator for another conversation. Immunisation coordinators felt that they were best placed to talk to parents who have concerns because of their “*experience in the area*,* our knowledge of what vaccine hesitancy is all about*,* it’s not*,* you know*,* it’s something where you have to work with the parent sensitively rather than just push your ideas onto them*” (Dianna).

The information participants shared with parents includes the Australian Immunisation Handbook (for the “*highly educated families who want more defined information*”), and the Sharing Knowledge About Immunisation (SKAI) website developed by Australian researchers [24]. The key informants also said that the SKAI website is regularly distributed through newsletters to GPs so that these professionals can share the resource with vaccine-hesitant parents.

Many participants were concerned about the “*awful*,” “*pervasive*,” “*overwhelming*” and “*frightening*” anti-vaccine information that parents may see on social media, and were particularly concerned about local anti-vaccine activists spreading misinformation:

*In quite a small community close by there’s a person with a very strong personality that is a vaccine refuser*,* and if you get one of those in a [social media] group they can spread their tentacles far –* Camille, workshop participant.

Some workshop participants were also concerned about the vaccine misinformation parents encounter in person. For instance, one workshop participant described an evangelical pastor *“providing incorrect information to his congregation…[regarding] the COVID vaccine*,* even the childhood vaccines and the flu vaccine”* (Melinda).

Despite their confidence in being the go-to people for vaccine concerns, participants remained quite unsure about how to handle the “*tentacles*” of anti-vaccine activists in regional and remote WA, which can result in parents refusing vaccines:

*We’re competing with a very simple blanket negative message*,* trying to counter that with a more complex scientific message is really difficult. And people get confused with*,* you know*,* which information’s right*,* it’s just easier to accept a very simple*,* don’t do it –* Martha, workshop participant.

#### Motivation

Despite the many obstacles that parents in regional and remote WA face when trying to vaccinate their children, workshop participants believed that the majority of parents are very motivated to overcome them. Some did report parental concern about the amount of vaccine needles given at the same time, but were apprehensive to space out vaccines given the access challenges: *“it just spreads things out, and the chances of them not coming back for whatever one they haven’t had is problematic” –* Beth, workshop participant.

### Previous strategies used to improve vaccine uptake in regional and remote WA

#### With parents

The most tried and tested strategy across all regions to improve vaccine uptake in children was information packs for parents. Over the years, packs have included vaccine schedule magnets, colouring-in books, pencils, pencil cases, stickers, balloons, bubbles, stick-on tattoos, and hats. They have been paid for with the incentive funds that immunisation providers (including WA Health) receive from the Federal Government for vaccinating children.

Some regions have accompanied this with mascots. ‘Superneedle’ was first introduced to one region in the 1990s. Local information packs were branded with ‘Superneedle’ and usually distributed to parents of children aged four-years. The community was involved in its development:

*…back in the late ‘90s*,* we did a colouring-in competition and one young chap at a primary school drew this gorgeous little character called ‘Superneedle’ for which he won the competition and from there we actually took that character and turned it into a real-life character…So*,* there is a number of costumes made that are around the region and we use that character to go out to schools*,* playgroups*,* daycare…to promote kids being vaccinated –* Sue, workshop participant.

Another region included the community to design a mascot. ‘Immy the Immunisation Echidna’ appears on the parental information pack. Other regions have either borrowed Immy’s costume for local events, or shown interest in replicating Immy to help improve vaccine uptake. However, an unpublished independent evaluation identified that although ‘Superneedle’ was accepted by parents and children, it did not appear to improve vaccine uptake directly. Audrey, a workshop participant, reported similar regrets regarding Immy:

*I wish to say [Immy] was a success*,* but I can’t say that because every time I look at the stats*,* they go up and down so…It’s popular… amongst parents as well as the staff; I think it’s a bit of reducing of the guilt that you jab or you administer five needles at the same time*,* at least you have something to offer.*

Parents of Aboriginal children in this region also receive books at certain ages that coincide with the vaccine schedule. Another strategy some regions have used, as referred to earlier, is the implementation of after-hours clinics to cater to families with busy lives. However, some have also trialled but ceased these clinics due to staffing issues and personal safety concerns. Beth, a workshop participant, had been campaigning for pharmacists to be able to vaccinate children with all vaccines as a strategy to improve access.

Other strategies have included outreach, reminders, home-visits, and bringing in families to the clinic using WA Health vehicles (all previously described). As described by a key informant, WAPHA also distribute a fridge magnet when the child is 6 weeks old with the vaccine schedule. One region’s WA Health office has a monthly vaccine information stand situated outside of shopping centres and/or grocery stores. Another region tried a tailored approach with parents of Aboriginal children. Families vaccinating children on time received baby clothes with ‘I’ve been immunised’ printed on the front. This reportedly elicited a mixed response, with some participants saying the local Aboriginal communities described it as a “*bribe*”. It is not clear what the impact of these strategies have been on vaccine uptake.

#### With immunisation providers and health care organisations

A strong sense of collegiality was evident among our participants as they aimed to help parents access vaccines on time. Participants work closely with the GP clinics and Aboriginal Medical Services in their area, as well as WAPHA. Roselyn (key informant from WAPHA) said they regularly distribute articles from NCIRS to those working in primary health in regional and remote WA. Audrey, a workshop participant, said staff from her unit regularly attend practice nurses’ meetings so that the nurses have the opportunity to ask questions about vaccination “*to increase their confidence and capacity in providing the immunisation service.”* This region’s office also provides quarterly immunisation newsletters to GP clinics about coverage and wastage. Gina (a workshop participant from another region) vaccinates attendees of ‘family days’ run twice a year by the local Aboriginal Medical Service. Gina also said of WAPHA:

*they do all our networking for us through all the GPs in [Town] and also throughout the region. So they are really an excellent source to help us to increase our coverage.*

Beth said in the workshop that her region also organises networking and upskilling events for immunisation providers to learn from vaccine hesitancy experts about how to talk to parents. The local WA Health team host similar events to help *“community health providers…become more proficient and more confident with their immunising skills.”*

Participants also meet regularly with their fellow immunisation coordinators to share ideas.

## Discussion

The barriers to immunisation of young children in regional and remote WA are clearly multifaceted; here we discuss participants’ views on strategies that could improve parents opportunities to vaccinate their children in regional and remote Western Australia (WA).

### Access

It needs to be easier for parents to physically access vaccines. Transport can be a challenge for parents, considering the weather and distance extremes experienced in regional and remote areas. In April 2022, the WA government introduced the ‘Maxi Vaxi’ bus which delivered COVID-19 vaccines to people in the Kimberley, Pilbara, Midwest and Goldfields [25]. While a permanent ‘Maxi Vaxi’ supplying routine vaccines to children in regional and remote WA may require more administrative and policy support, it could help to improve uptake. Conversely, some participants did speculate that providing significant aid and outreach may disempower and unintentionally discourage some parents from actively organising their children’s vaccinations. As such, it is necessary to determine whether the offer of transport delivers empirical results through surveys or formal trials.

It is also apparent that increasing the number of walk-in and after-hours appointments would help parents. Despite some regions ceasing after-hour clinics, even participants with concerns about the safety of immunisation providers felt that having more clinics with flexible hours would help parents access vaccines in a timely manner.

Flexible vaccination options appear to be particularly desirable and culturally appropriate for Aboriginal families, as well as refugee families who want or need the fathers’ involvement in the vaccination process. This finding, however, is not unique to WA: in regional New South Wales, on the east coast of Australia, more flexible options (e.g., drop-in services) were also required [26]. To facilitate this locally, WA Health should consider creating protocols by which staff are contracted to work “after-hours” time slots. Relatedly, it is well-established that there are staffing issues in the regions for a range of reasons, including a nation-wide healthcare worker shortage and concerns for personal safety [26–28]. Ensuring that staff feel safe, and are available to vaccinate on the day that reminder phone calls are made, and ensuring that every nurse on staff has a vaccination certificate will help improve the efficiency of walk-ins.

### Workforce strength and support

Participants reflected that many immunisation providers lack the skillset to discuss vaccination with vaccine hesitant parents. All providers should receive training on how to confidently converse with vaccine-hesitant parents. Sharing Knowledge About Immunisation (SKAI) is an evidence-based (albeit, to the best of our knowledge, yet to be formally evaluated) toolbox for providers to use for confident vaccine communication with patients [24], and could be more widely promoted by WA Health and utilised among immunisation providers.

Additionally, though uptake of vaccines in Aboriginal children is high in some regions, there is room for improvement in others. Aboriginal Health Workers (AHWs) and Aboriginal Health Practitioners (AHPs – individuals who meet requirements to be registered with the Aboriginal and Torres Strait Islander Health Practice Board of Australia) need further support to confidently administer vaccines to these children. A Cochrane review identified that promotion of immunisation by “lay” health workers (such as AHWs) can increase immunisation [29], so physically being able to administer the vaccine in addition to promoting it would likely see an increase in vaccine uptake. In WA, AHWs who complete an immunisation course can vaccinate under medical supervision, but, according to our workshop participants, many are still uncomfortable doing so, particularly multiple doses. WA Health, recognising the positive impact AHPs can have on immunisation services for the WA Aboriginal community, enacted in March 2024 a Structured Administration and Supply Arrangements, referred to as a SASA. This is a written direction that authorises AHPs trained in immunisation (through approved immunisation courses) and working in WA Health clinics, the Department of Justice, and a health service that is a member of the Aboriginal Health Council of WA to administer immunisations according to the WA immunisation schedule [30]. Now that AHWs and AHPs are afforded the ability to vaccinate their patients, and receiving education that improves their confidence, vaccination coverage in Aboriginal communities across WA may improve.

Finally, some participants described how the GPs they interact with range from hesitant to reluctant to navigate complex immunisation schedules for individuals overdue for vaccination as per Australia’s NIP. It is important to note that developing a ‘catch up’ immunisation plan is complex and time consuming. Where possible, workshop participants are providing 1-on-1 support to GPs however it was described as a “hard” task; further support from WA Health to navigate these complexities is required. This may involve promotion of the Australian Government’s online ‘Catch-up Calculator’ which assists immunisation providers to plan an appropriate catch-up schedule for children aged < 10 years [31].

### Reminders

Governments need to implement automated reminder systems for parents of 18-month-old children. A meta-analysis of 120 studies identified that reminders can improve childhood vaccine uptake by 15% [32]. The removal of the 18-month health check in WA was identified by participants as a major barrier as parents aren’t receiving prompts about upcoming vaccines. Given how much time our participants reported spending going through their local vaccine uptake data, and then sending out tailored SMS reminders, the reminder system needs to be automated – although the ability to do so is contingent on having accurate data.

A popular strategy among participants was a mobile app with vaccine reminders and child health information for the “*technology savvy young parents*.” Participants said these were available for parents in other Australian states. Having an app removes the barrier of phone numbers and home addresses being inaccurate in an immunisation provider’s database, but does rely on the parent initially downloading it onto their phone, having a decent level of digital literacy, not be living with digital poverty, and for the phone to be solely used by the carer/s responsible for vaccination and not shared among families (a barrier identified by Bolsewicz et al. for regional families in New South Wales [26].

### Limitations and future work

This is the first study to systematically seek to understand why two-year-old children in regional and remote WA have suboptimal vaccine uptake. Though we only sought to understand WA, given that the national vaccination rate dips at the 24-month mark, it is likely that there are common barriers at the national level. However, in this study we have uncovered some local suggestions that WA Health can investigate. Our study, however, is not without limitations. For example, due to the pressures of COVID-19 and its vaccine rollout, we were unable to recruit anyone from one particular region. However, informants from WA Health indicated that the issues mentioned by those who did attend the workshop are issues that pertain to that region too. Further, this study did not investigate the perspectives of parents; this is the next stage of our research. Relatedly, considering the suggestion that providers should not excessively cater to parents, we also plan to examine questions of systemic versus individual agency.

## Conclusion

Through a combination of semi-structured interviews and focus groups, this study has investigated the views of key immunisation stakeholders in the state of Western Australia, aiming to better understand the factors influencing – and possible solutions to – sub optimal childhood vaccine uptake for toddlers in regional and remote areas. While childhood under-vaccination is of global importance, this research was specifically motivated by vaccination rates for WA children aged 24 months being lower than at 12 and 60 months.

The findings suggest that a multicomponent intervention is required. Participants described facing a range of challenges, which local health workers and public agencies have attempted to mitigate on an often ad-hoc basis. These include lack of parental awareness of the vaccine schedule, difficult access to services, a shortage of experienced health workers able to converse meaningfully with vaccine hesitant parents, ineffective reminder systems, challenges in data management and governance, and the rapid spread of misinformation. Workforce support – particularly for Aboriginal Health Workers – improved reminder systems and additional efforts at improving access to health services are plausible and likely effective interventions available to government agencies in Western Australia.

## Data Availability

The datasets generated and/or analysed during the current study are not publicly available due to ethics restrictions, but are available from the corresponding author on reasonable request.
